# Fabrication of black NiO/Sr_2_FeTaO_6_ heterojunctions with rapid interface charge transfer for efficient photocatalytic hydrogen evolution

**DOI:** 10.3389/fchem.2022.1118540

**Published:** 2023-01-06

**Authors:** Guiyun Yu, Jiawei Hu, Wen Xiao, Yongtai Zhu, Yong Dai

**Affiliations:** ^1^ School of Chemistry and Chemical Engineering, Yancheng Institute of Technology, Yancheng, China; ^2^ School of Materials Science and Engineering, Yancheng Institute of Technology, Yancheng, China; ^3^ Tianneng Carbon Co., Ltd, Yancheng, China

**Keywords:** photocatalysis, Sr_2_FeTaO_6_, NiO, hydrogen evolution, heterojunction

## Abstract

Series of black NiO/Sr_2_FeTaO_6_ (NiO/SFT) composites were synthesized by the combined processes of hydrothermal method and calcination treatment. The formed NiO was deposited on the surface of Sr_2_FeTaO_6_ to form a closely interfacial contact, leading to the formation of NiO/Sr_2_FeTaO_6_ heterojunction. The resulted samples were fully characterized by XRD, TEM, XPS, and UV-Vis DRS to gain their microstructure, crystal phase, atomic states and optical absorption properties. Introducing narrow-bandgap semiconductor of black NiO in NiO/Sr_2_FeTaO_6_ heterojunctions exhibits two major advantages. On the one hand, coupling with black NiO can significantly increase the light harvesting capacity of Sr_2_FeTaO_6_. On the other hand, the formed NiO/Sr_2_FeTaO_6_ heterojunctions benefited the separation and transfer of photogenerated charge carriers, which was confirmed by photo-electrochemical measurement, PL and TR-PL spectra. The activity of as-prepared samples was evaluated by photocatalytic hydrogen (H_2_) evolution (PHE) under visible light irradiation. The resulted NiO/SFT composites showed the improved PHE efficiency than that of NiO and Sr_2_FeTaO_6_, owing to the synergistic effects of synergistic effects of heterojunction formation for the efficient charge carrier transfer/separation and increased light harvesting capacity. However, the excess amount of NiO loaded in NiO/SFT composites will restrain the light harvesting of Sr_2_FeTaO_6_ component and decrease, leading to the decreased PHE activity. Our work provided an insight on the construction of high-efficiency heterojunction photocatalysts for PHE reaction.

## 1 Introduction

Recently, photocatalytic H_2_ evolution (PHE) driven by solar energy has been extensively studied and regarded as an effective approach to solve the problems of the energy shortage crisis and environmental pollution (Wang and Domen, 2020; Wang et al., 2021a; Zhang et al., 2022). Up to now, various semiconductor photocatalysts have been developed for H_2_ evolution, including CdS (Cheng et al., 2018), ZnIn_2_S_4_ (Wang et al., 2021b; Liu et al., 2023), layered double hydroxide (LDH) (Boumeriame et al., 2022), metal oxides (Liu et al., 2020), graphitic carbon nitride (g-C_3_N_4_) (Ong et al., 2016), perovskites (Mai et al., 2021), TiO_2_ (Chen and Mao, 2007), and so on. As a traditional photocatalyst, TiO_2_ has been widely investigated due to its low cost, toxicity, excellent chemical stability, high photo-activity and suitable optical and electronic properties (Chen et al., 2010). Nevertheless, the limited UV light absorption capacity and rapid charge carrier recombination rate restrain the practical applications of TiO_2_ material. Therefore, we will devote much effort to develop novel visible light-driven PHE catalysts with a high efficiency.

Among the reported photocatalysts, metal oxides with a perovskite-type structure (ABO_3_) often present intriguing photocatalytic activity (Wagner and Somorjai, 1980; Ham et al., 2016; Jiang et al., 2020; Li et al., 2022a). Especially, Ta-contained perovskite oxides, such as NaTaO_3_, are efficient photocatalysts for H_2_ evolution due to their strong redox ability (Kudo and Kato, 2000; Wang et al., 2020). By altering internal constituent elements of ABO_3_-type perovskite, different physicochemical properties can be adjusted so as to meet the demands of diverse applications. Specially, the double-perovskite compound Sr_2_FeTaO_6_ has attracted increasing attention in photocatalytic field due to the fact that the introduction of Fe element in the structure by partially filling *d* orbitals can widen visible-light response region for enhancing photocatalytic activity (Chen and Xu, 2017). However, the large bandgap value and rapid charge carrier recombination over pure Sr_2_FeTaO_6_ have restricted its photocatalytic efficiency.

Various modified methods, such as heterojunction formation (Liu et al., 2018; Guan et al., 2019), elemental doping (Wang et al., 2019; Zhang et al., 2021), morphology engineering (Liu et al., 2015), noble-metal loading (Liu et al., 2022a), have been regarded as the potential approaches to solve the above mentioned drawbacks. For example, (Cui et al., 2019) developed a facile hydrothermal method to prepare Sr_2_FeTaO_6_/NaTaO_3_ heterojunctions by partly transforming NaTaO_3_ sheets into Sr_2_FeTaO_6_ component (Cui et al., 2019). The formed Sr_2_FeTaO_6_/NaTaO_3_ heterojunctions with a closely interfacial contact showed the enhanced visible-light-driven photocatalytic performance for NO reduction and H_2_ generation due to the rapid transfer and separation of photogenerated charge carriers. Thus, constructing heterojunction photocatalysts by coupling with narrow-bandgap semiconductor have been proved to be an effective solution due to dual advantages of the efficient charge carrier transfer and separation and increased light harvesting capacity.

Recently, NiO material has been widely investigated and acted as co-catalyst in the photocatalytic water splitting due to its unique electronic structure. This intriguing 3*d* electronic structure of NiO is localized in space but distributed in a wide energy region due to its strong mutual coulomb repulsion, leading to a high mobility of photo-induced charge carriers in NiO (Luo et al., 2015; Wang et al., 2017). Especially, novel black NiO nanoparticle, with lattice defects and intermediate energy level, has a profoundly narrowed bandgap value of ∼1.42 eV (Wang et al., 2017). Up to now, no attempt has been made for combining black NiO with Sr_2_FeTaO_6_ for photocatalysis.

In light of the above consideration, this work employed a facile hydrothermal process to synthesize black NiO/Sr_2_FeTaO_6_ (NiO/SFT) composites with a closely interfacial contact. The activity of as-prepared samples was evaluated by PHE reaction under visible light irradiation. The introduction of black NiO in NiO/SFT composites exhibited two advantages of increased light harvesting capacity and enhanced charge carrier separation and transfer, leading to the high PHE efficiency. A possible PHE mechanism was proposed based on the experimental results.

## 2 Materials and methods

### 2.1 Preparation of Sr_2_FeTaO_6_

To prepare Sr_2_FeTaO_6_, Sodium oleate (300 mg) was firstly dissolved in a mixture of ethanol (52.5 mL) and oleic acid (7.5 mL) under constant stirring. Then ferric acetylacetonate (1.059 g), strontium acetate (1.234 g) and tantalum pentachloride (1.074 g) were separately added into above solution with a sealed condition. After stirring for 30 min, 15 mL sodium hydroxide (.5 M) was rapidly added and then a faint yellow emulsion appeared. After reaction for 60 min, the obtained faint yellow solution was transferred into a 100 mL Teflon-lined autoclave and heated at 200°C for 12 h. After cooling to room temperature, the obtained precipitates were gathered by centrifuged (6,000 r/min), washed with ethanol and deionized water several times, and subsequently dried under vacuum at 60°C to gain Sr_2_FeTaO_6_ powder.

### 2.2 Preparation of black NiO nanoparticles

The black NiO nanoparticles were prepared by a two-step approach containing hydrothermal method and subsequent calcination treatment. Nickel nitrate (5 mmol) and urea (15 mmol) were dissolved in mixed solution of 50 mL deionized water and 25 mL ethanol. After ultrasonic treatment for 30 min, aforementioned solution was transferred into a 100 mL Teflon-lined autoclave and heated at 120°C for 12 h. The obtained NiO precursor was washed with deionized water and ethanol several times, and then dried in a vacuum oven at 60°C. Finally, NiO precursor was directly calcined at 500°C for 2 h in air to gain black NiO nanoparticles.

### 2.3 Synthesis of black NiO/Sr_2_FeTaO_6_ (NiO/SFT) composites

*x* mmol Ni(NO_3_)_2_·6H_2_O (*x* = 1, 3, 5, 7, 9), (10-*x*) mmol Sr_2_FeTaO_6_ powders, and 15 mmol urea were added into the mixture of deionized water (50 mL) and ethanol (25 mL) under the continuous ultrasonic treatment for 30 min. The remained process for synthesizing *x*NiO/(10-*x*) SFT composites was similar to that of NiO. For comparison, black NiO nanoparticles were simply mixed with Sr_2_FeTaO_6_ to prepare the mixed sample of 3NiO/7SFT (MIXT), showing the same composition with 3NiO/7SFT.

### 2.4 Characterization

The morphology, phase structure and composition of the samples were obtained by various characterization techniques. High-resolution transmission electron microscope (HRTEM) was operated on JEOL JEM-200CX to measure the microstructure and lattice fringes of photocatalysts. X-ray diffraction pattern (XRD) was collected on a powder X-ray diffractometer (XRD, Philip) using Cu Kα radiation (*λ* = 1.5408Å). The X-ray photoelectron spectroscopy (XPS) measurement was performed in a VG Scientifific ESCALAB250-XPS photoelectron spectrometer with an Al Kα X-ray (*hν* = 1486.6 eV) source. UV–Vis diffuse reflection spectroscopy (DRS) was obtained on a HITACHI U-3310 spectrophotometer to investigate the optical properties of the samples. Photoluminescence (PL) spectra were recorded on JASCO FP 6500 fluorescence spectrometer with using a He-Cd laser source in the excitation wavelength. Time-resolved photoluminescence (TR-PL) decay spectra of as-prepared samples were measured by a F900 fluorescence spectrometer with an excitation wavelength of 310 nm.

### 2.5 Photocatalytic H_2_ evolution measurement

The photocatalytic performance of as-synthesized samples was evaluated by measuring H_2_ evolution amout under visible light. During PHE experiment, a 300 W Xenon lamp (PLS-SXE300, *λ* > 420 nm) was used as visible light source. In the H_2_ production experiment, 50.0 mg photocatalyst was dispersed into the aqueous solution containing triethanolamine (TEOA, 20vol%) as the hole sacrificial reagent and H_2_PtCl_6_ solution (2 wt% Pt) as cocatalyst. Prior to PHE reaction, the reactor system was vacuumed by using a vacuum pump to remove air completely. The amount of generated H_2_ was determined by gas chromatography (Shimadzu, GC-2014).

## 3 Results and discussion

**FIGURE 1:**
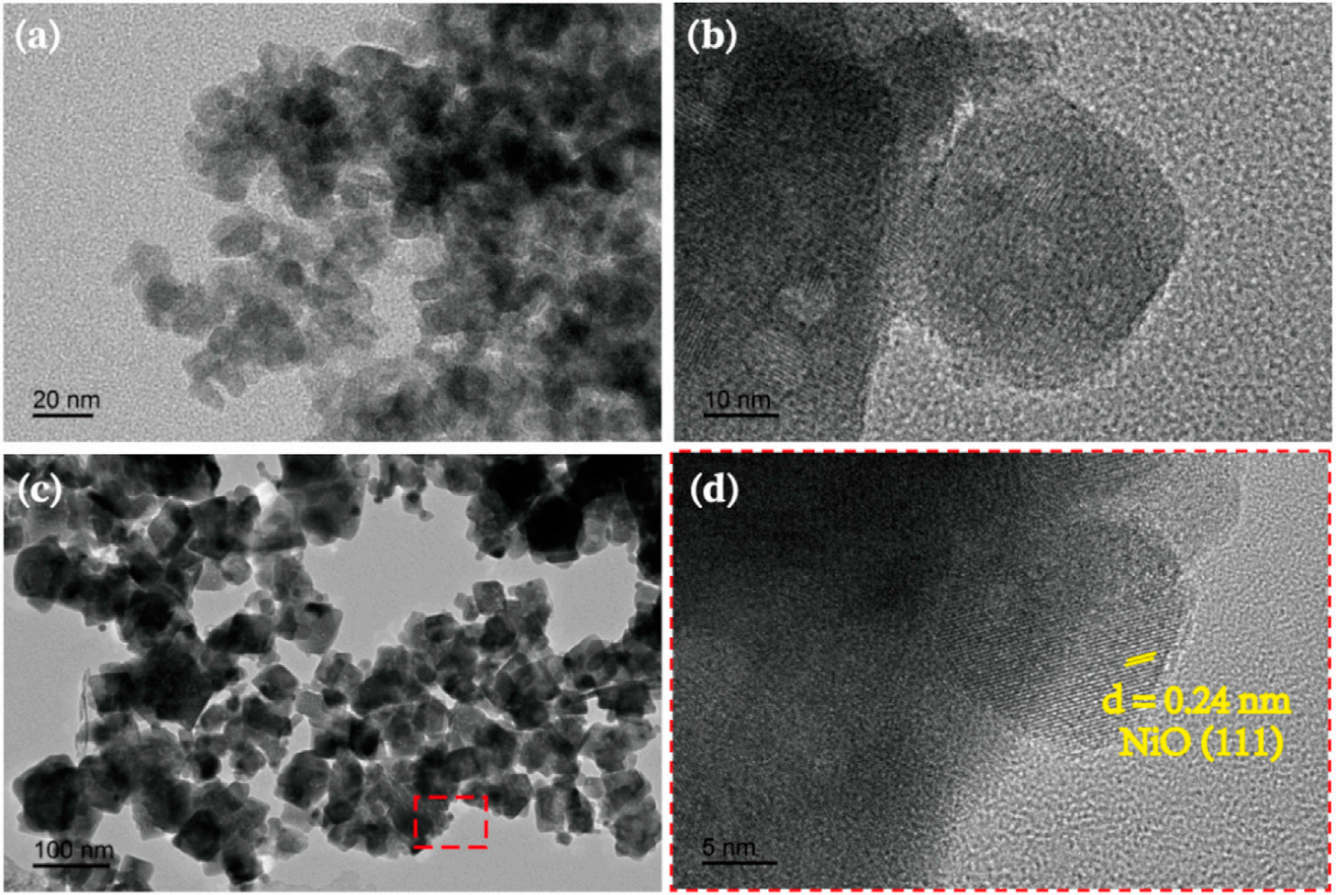
TEM images of **(A)** black NiO nanoparticles, **(B)** Sr_2_FeTaO_6_ and **(C)** 3NiO/7SFT. HRTEM image of **(D)** 3NiO/7SFT.

To confirm the formation of heterojunction between NiO and Sr_2_FeTaO_6_, HRTEM images were conducted and presented in Figure 1. The TEM image of NiO shows the clear nanoparticle-like morphology with an average diameter of ∼20 nm and accumulated pattern (Figure 1A). Compared with NiO, Sr_2_FeTaO_6_ sample has the similar particle morphology but a larger particle size (Figure 1B). After coupling NiO with Sr_2_FeTaO_6_, the resulted 3NiO/7SFT exhibits the clearly accumulated nanoparticles with an increased average diameter of ∼50 nm. From the enlarged area (red line) in Figure 1C, there is an interplanar spacing of lattice fringes of .24 nm, corresponding to the (111) planes of NiO (John et al., 2022). It can be observed that NiO nanoparticles are deposited on the surface of Sr_2_FeTaO_6_, leading to the formation of heterojunction. This formed NiO/Sr_2_FeTaO_6_ heterojunction benefits the charge carrier separation and transfer, thereby boosting PHE efficiency (Yu et al., 2022a; Zeng et al., 2022).

**FIGURE 2:**
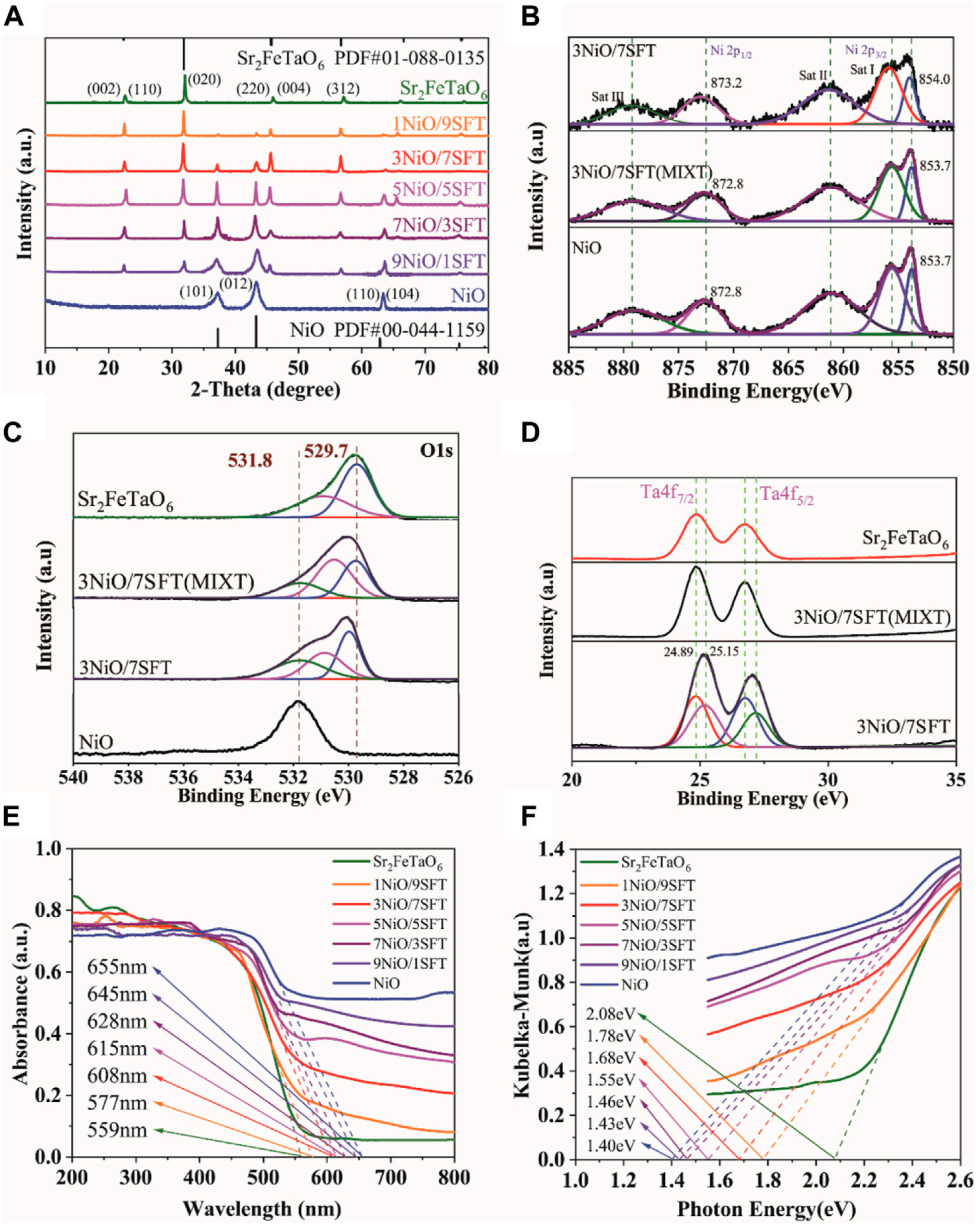
**(A)** XRD patterns of NiO, Sr_2_FeTaO_6_ and *x*NiO/(10-*x*)SFT composites (*x* = 1, 3, 5, 7, and 9). XPS spectra of NiO, Sr_2_FeTaO_6_, 3NiO/7SFT and 3NiO/7SFT(MIXT): **(B)** Ni 2p, **(C)** O 1s and **(D)** Ta 4f. **(E)** UV–vis DRS of NiO, Sr_2_FeTaO_6_ and *x*NiO/SFT (*x* = 1, 3, 5, 7, and 9) composites and **(F)** their corresponding Tauc plots.

The X-ray diffraction (XRD) patterns of as-prepared samples were measured to determine their crystal structure and composition. As shown in Figure 2A, the characteristic peaks of obtained Sr_2_FeTaO_6_ and NiO are in agreement with the published data of PDF#01-088-0135 and PDF#00-044-1159, respectively, confirming their successful preparation. Furthermore, the resulted *x*NiO/SFT (*x* = 1, 3, 5, 7, and 9) composites exhibit the dual characteristic signals of both NiO and Sr_2_FeTaO_6_. With the increasing of NiO loading amount, the peak intensity of NiO is gradually increased. Thus, it indicates that NiO is successfully coupled with Sr_2_FeTaO_6_ to form NiO/Sr_2_FeTaO_6_ heterojunction.

The chemical composition and atomic states of as-prepared samples were studied by X-ray photoelectron spectroscopy (XPS) (Li et al., 2022b). Figure 2B shows the Ni 2p XPS spectra of NiO, 3NiO/7SFT (MIXT) and 3NiO/7SFT. The characteristic peaks of NiO at 853.7 and 872.8 eV belong to Ni 2p_3/2_ (Ni^2+^) and Ni 2p_1/2_ (Ni^3+^), respectively (Liu et al., 2022b). Three satellite bands of Sat I, Sat II and Sat III are visible in NiO. Compared with NiO sample, 3NiO/7SFT (MIXT) shows an unchanged peak position, while a slight positive shift for 3NiO/7SFT sample, indicating the presence of a strong interaction between NiO and Sr_2_FeTaO_6_.

In the XPS spectra of O 1 s (Figure 2C), two characteristic peaks of Sr_2_FeTaO_6_ at 529.7 eV and 531.8 eV are attributed to lattice Ta-O-Fe and surface oxygen (O_sur_), respectively, while only one characteristic peak of O_sur_ at 531.8 eV is visible in NiO (Cui et al., 2019; Pan et al., 2022). In comparison with pure NiO, the peak position at ∼529.7 eV derived from lattice Ta-O-Fe is maintained in 3NiO/7SFT (MIXT) sample while exhibit a positive shift for 3NiO/7SFT. Furthermore, the XPS spectrum of Ta 4f shows two characteristic peaks at 24.89 eV (Ta 4f_7/2_) and 25.15 eV (Ta 4f_5/2_) in Sr_2_FeTaO_6_ (Figure 2D). The position of these two characteristic peaks is almost unchanged in 3NiO/7SFT (MIXT) but shifted positively in 3NiO/7SFT sample, further confirming the existence of strong interaction between NiO and Sr_2_FeTaO_6_.

The UV-Vis diffuse reflectance spectra (UV-Vis-DRS) were examined to investigate the optical absorption properties of as-prepared samples (Figure 2E). The light absorption edge means the intercept between the tangent of the absorption plot and the X-axis. Bulk NiO exhibit a strong absorption capacity in the visible light region with an absorption edge at 655 nm, while a relatively low visible light harvesting ability is visible in pure Sr_2_FeTaO_6_ with an absorption edge at 559 nm. After coupling NiO with Sr_2_FeTaO_6_, the resulted *x*NiO/(10-*x*)SFT composites (*x* = 1, 3, 5, 7, and 9) show widened absorption regions and increased light absorption edge values from 577 nm for 1NiO/9SFT to 645 nm for 9NiO/1SFT, which are strongly dependent on NiO deposition amount. Additionally, the bandgap values of various samples are resulted from the UV–Vis DRS absorption plots (Figure 2F). According to the Kubelka-Munk function transformation, the bandgap values of Sr_2_FeTaO_6_, 1NiO/9SFT, 3NiO/7SFT, 5NiO/5SFT, 7NiO/3SFT, 9NiO/1SFT and NiO are estimated to be 2.08, 1.78, 1.68, 1.55, 1.46, 1.43, and 1.40 eV, respectively (Yu et al., 2022b). This result indicates that the deposition of NiO can significantly increase light harvesting capacity of Sr_2_FeTaO_6_ in NiO/SFT composites, thus reducing bandgap values.

**FIGURE 3:**
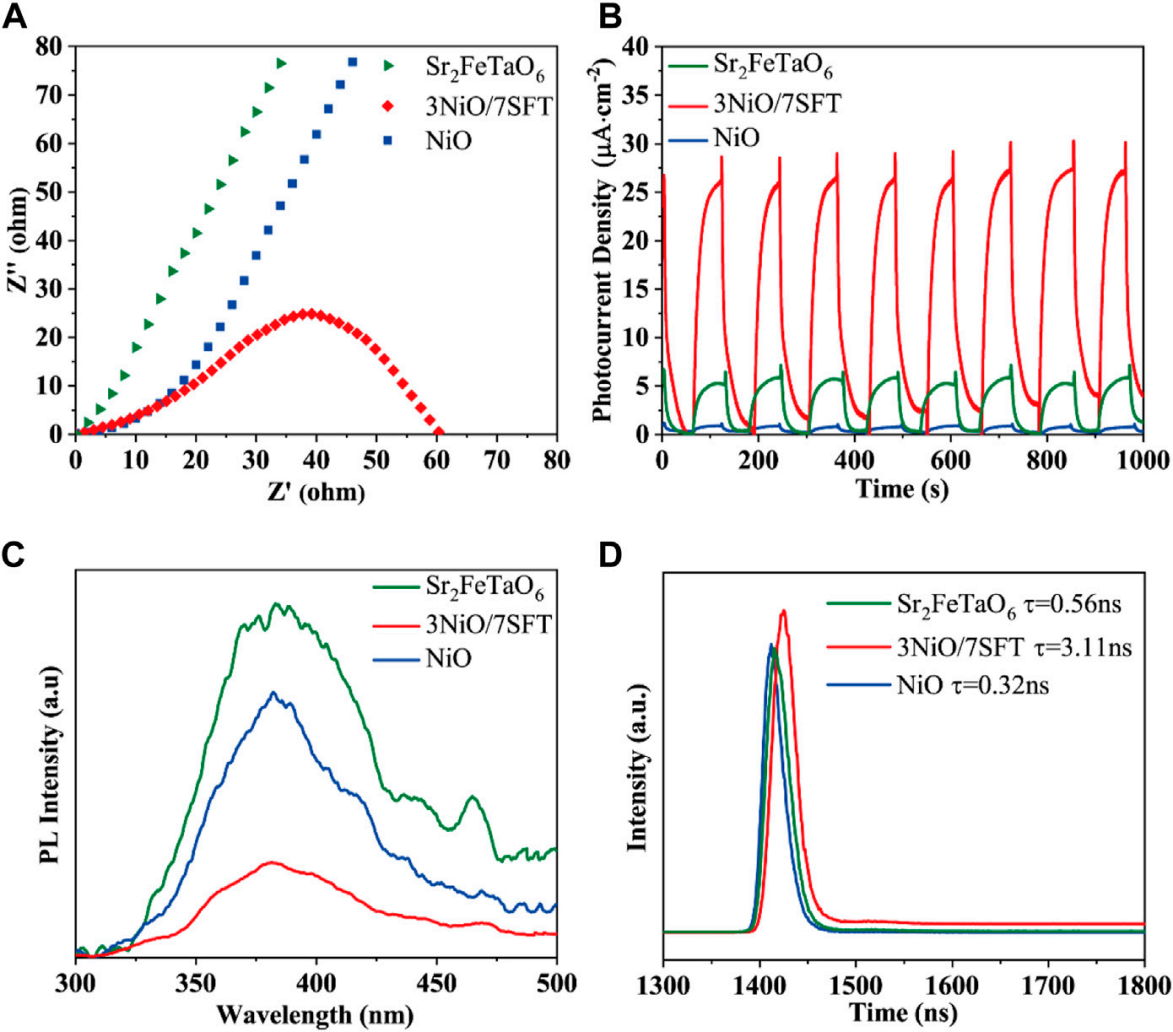
**(A)** Electrochemical impedance spectra, **(B)** transient photocurrent responses, **(C)** steady-state PL spectra and **(D)** TR-PL decay spectra of NiO, Sr_2_FeTaO_6_, and 3NiO/7SFT.

The electrochemical impedance spectroscopy (EIS) and transient photocurrent response of as-prepared samples were performed to analyze charge carrier migration and separation rate (Yu et al., 2020; Liu et al., 2021a). Figure 3A displays the EIS plots of NiO, Sr_2_FeTaO_6_, and 3NiO/7SFT with a typical semicircular arc. The diameter of the semicircular arc can indirectly reveal the migration and separation mechanism of photogenerated electrons and holes (Li et al., 2022c). A lower charge transfer resistance means a higher charge carrier separation efficiency (Wang et al., 2022). Specially, the semicircle diameter of 3NiO/7SFT composite is smaller than that of NiO and Sr_2_FeTaO_6_, indicating that the formation of NiO/Sr_2_FeTaO_6_ heterojunction is beneficial to the separation and transfer of photogenerated charge carriers.

The transient photocurrent responses of NiO, Sr_2_FeTaO_6_, and 3NiO/7SFT were measured under visible light for eight cycles (Figure 3B). Clearly, the transient photocurrent responses of NiO, Sr_2_FeTaO_6_, and 3NiO/7SFT all show high repeatability. Under visible light, the photocurrent intensity of 3NiO/7SFT sample is higher than that of NiO and Sr_2_FeTaO_6_. It demonstrates that the photo-induced electrons and holes are efficiently separated in 3NiO/7SFT composite due to the formation of NiO/Sr_2_FeTaO_6_ heterojunction, significantly boosting photocatalytic efficiency for H_2_ evolution.

Steady-state photoluminescence (PL) spectroscopy was developed to investigate the recombination capacity of the photogenerated electrons and holes in as-prepared samples (Liu et al., 2017; Liu et al., 2019). The PL intensity is proportional to the charge carrier recombination rate. The PL spectra of NiO, Sr_2_FeTaO_6_ and 3NiO/7SFT at an excitation wavelength of 315 nm are shown in Figure 3A. The PL intensity of 3NiO/7SFT sample is profoundly restrained, corresponding to the reduced recombination efficiency of charge carrier. Therefore, the effective separation of photogenerated electrons and holes in 3NiO/7SFT sample benefits the enhanced photocatalytic activity. Time-resolved PL (TR-PL) decay spectra of photocatalysts were characterized to reveal their charge transfer kinetics (Liu et al., 2021b; Liu et al., 2021c). Compared to NiO (*τ* = .32 ns) and Sr_2_FeTaO_6_ (*τ* = .56 ns), 3NiO/7SFT (*τ* = 3.11 ns) sample has the increased average emission lifetime (Figure 3B), indicating that the formed NiO/Sr_2_FeTaO_6_ heterojunction can significantly hinder the recombination of photogenerated electron-hole pairs, thus enhancing photocatalytic activity for H_2_ evolution.

**FIGURE 4:**
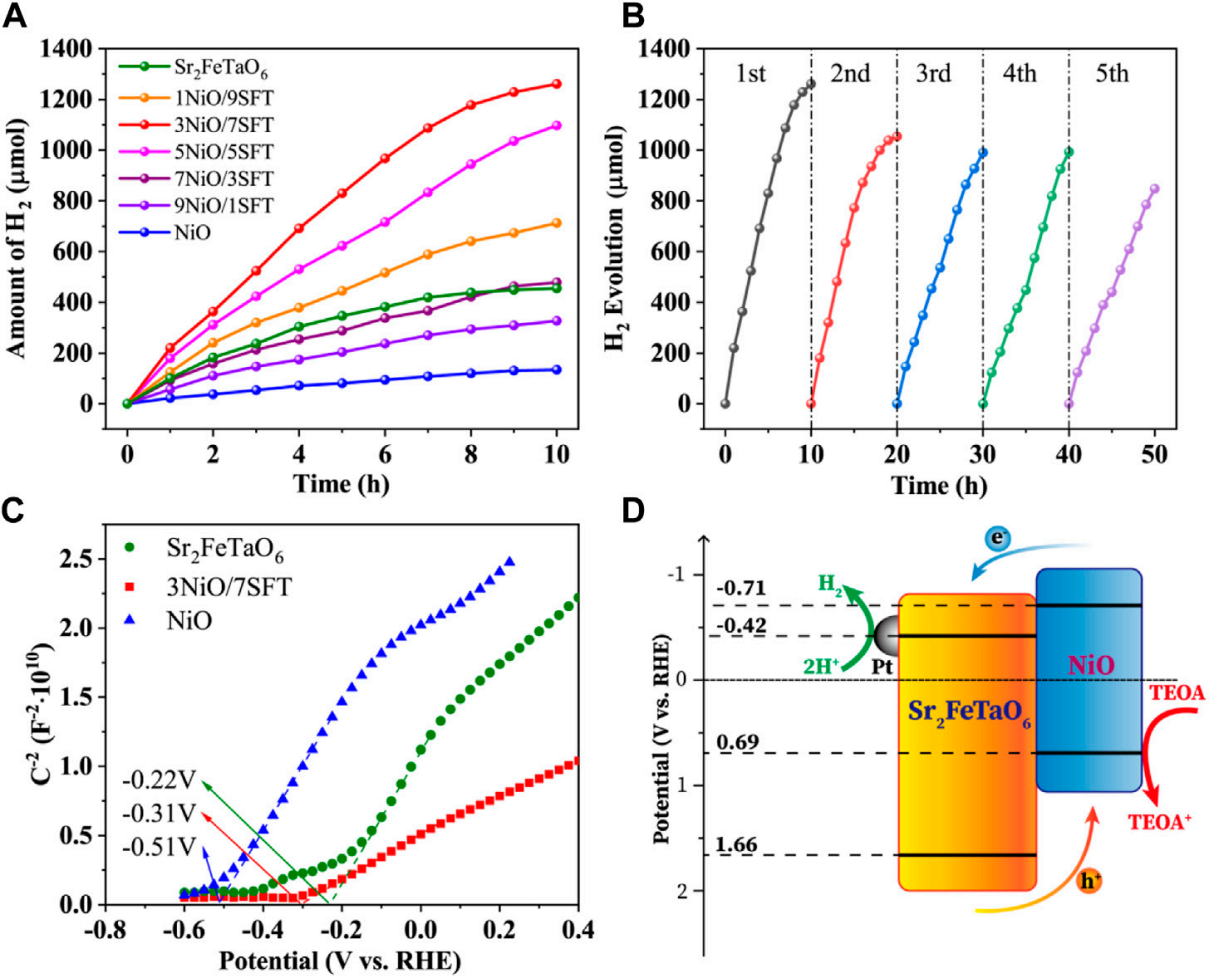
**(A)** Photocatalytic H_2_ production activities of NiO, Sr_2_FeTaO_6_, and *x*NiO/(10-*x*)SFT composites (*x* = 1, 3, 5, 7, and 9). **(B)** Recyclability study of 3NiO/7SFT for five successive photocatalysis cycles. **(C)** Mott-Schottky plots for NiO, Sr_2_FeTaO_6_, and 3NiO/7SFT. **(D)** Schematic illustration of charge transfer and H_2_ evolution mechanism over 3NiO/7SFT photocatalyst.

The photocatalytic H_2_ evolution performance of NiO, Sr_2_FeTaO_6_ and *x*NiO/(10-*x*)SFT composites (*x* = 1, 3, 5, 7, and 9) was evaluated under visible light using TEOA aqueous solution as sacrificed electron donor. As shown in Figure 4A, the photocatalytic H_2_ evolution of NiO and Sr_2_FeTaO_6_ exhibit the relatively low rates of 268.9 and 910.9 μmol h^−1^ g^−1^, owing to the rapid recombination of photogenerated electrons and holes. In the *x*NiO/(10-*x*)SFT (*x* = 1, 3, 5, 7, and 9) photocatalytic system, the hydrogen production rate is increased with the increasing of NiO loading amount, showing the optimal example of 3NiO/7SFT with the maximum H_2_ evolution efficiency of 2,944 μmol h^−1^ g^−1^. With the loading amount increasing of NiO, the overall hydrogen production rates are correspondingly decreased. It demonstrates that the synergistic effects of heterojunction formation and increased light harvesting capacity play a vital role in boosting photocatalytic activity. However, the excess loading amount of NiO will inhabit the light absorption of Sr_2_FeTaO_6_ component and decrease the overall light utilization efficiency, leading to the reduced photocatalytic activity. In order to evaluate photo-stability, the photocatalytic H_2_ evolution experiment of 3NiO/7SFT sample was carried out for five cycles under the same condition (Figure 4B). The PHE rate is gradually reduced over time, owing to the consumption of sacrificial agent and slight loss of photocatalyst. After five cycles, the average H_2_ generation efficiency still remains 1695.0 μmol h^−1^ g^−1^ after 50 h under visible light irradiation, demonstrating that 3NiO/7SFT sample shows the relatively high photo-catalytic stability.

The conduction band (CB) positions of Sr_2_FeTaO_6_, 3NiO/7SFT and NiO were calculated based on Mott-Schottky (MS) curves (Figure 4C). The positive slope of the curves reveals their n-type semiconductors. The flat band potentials of Sr_2_FeTaO_6_, 3NiO/7SFT and NiO are determined to be −.22 V, −.31 V, and −.51 V (vs. RHE), respectively, by the intercept of X axis (potential axis). According to the n-type semiconductor theory, the flat band potential is .2 V higher than CB potential (*E*_CB_). Thus, the *E*_CB_ of Sr_2_FeTaO_6_, 3NiO/7SFT and NiO are calculated to be −.42 V, −.51 V, and −.71 V (vs. RHE), respectively. Combining the bandgap values of Sr_2_FeTaO_6_ (2.08 eV) and NiO (1.40 eV), the CB and valance band (VB) potentials of Sr_2_FeTaO_6_ and NiO are calculated to be −.42 V/+1.66 V (vs. RHE) and −.71 V/+.69 V (vs. RHE), respectively.

A possible PHE mechanism was proposed as follows. Under visible light, both Sr_2_FeTaO_6_ and NiO are excited to generate electrons and holes. The generated electrons in CB of NiO will migrate to CB of Sr_2_FeTaO_6_ and then to Pt nanoparticles for photocatalytic H_2_ evolution reaction, while holes will oppositely transfer from VB of Sr_2_FeTaO_6_ to VB of NiO across the intimate interface due to the potential difference. The accumulated holes will be consumed by the sacrificial regent. The enhanced photocatalytic activity for H_2_ evolution is ascribed to the synergistic effects of heterojunction formation and increased light harvesting capacity.

## 4 Conclusion

In this work, a simple hydrothermal method was firstly employed to prepare Sr_2_FeTaO_6_ nanoparticles, and then fabricated series of black NiO/Sr_2_FeTaO_6_ (NiO/SFT) composites by a two-step process containing hydrothermal method and calcination treatment. The formed NiO was deposited on the surface of Sr_2_FeTaO_6_ to form a closely interfacial contact, resulting in the heterojunction formation between NiO and Sr_2_FeTaO_6_. Based on photo-electrochemical measurement, PL and TR-PL spectra, the formed NiO/Sr_2_FeTaO_6_ heterojunctions are beneficial to the charge carrier separation and transfer. The resulted NiO/SFT composites showed the higher PHE activity than that of NiO and Sr_2_FeTaO_6_, and the optimal sample of 3NiO/7SFT has the highest PHE efficiency of 2,944 μmol h^−1^ g^−1^. The enhanced PHE performance was ascribed to the synergistic effects of synergistic effects of heterojunction formation for the efficient charge carrier transfer/separation and increased light harvesting capacity. Notably, the excess loading amount of NiO in NiO/SFT composites will result in the reduced PHE activity due the decreased light harvesting capacity toward Sr_2_FeTaO_6_ component. Our work provided an insight on the development of high-efficiency heterojunction photocatalysts for PHE reaction by introducing narrow-bandgap semiconductor.

## Data Availability

The original contributions presented in the study are included in the article/supplementary material, further inquiries can be directed to the corresponding authors.
